# Relationship Between Personality Traits and Workplace Bullying Victims in Saudi Arabia

**DOI:** 10.7759/cureus.58474

**Published:** 2024-04-17

**Authors:** Khalid M Alduraibi, Ali I Alfarhan, Deemah M Alghaith, Abdulrahman D Alharbi, Mohammad S Almosa

**Affiliations:** 1 College of Medicine, King Saud Bin Abdulaziz University for Health Sciences, Riyadh, SAU; 2 Family Medicine, King Abdulaziz Medical City College of Medicine, King Saud Bin Abdulaziz University for Health Sciences, King Abdullah International Research Center, Riyadh, SAU

**Keywords:** workplace, saudi arabia, job performance, bullying, personality traits

## Abstract

Background

Workplace bullying is persistent aggressive behavior, including verbal or physical abuse, exhibited in a working environment. The impact of workplace bullying in any industry leads to negative outcomes in multiple dimensions, such as issues with mental health, problems with physical health, and a reduction in productivity in the workplace. This study aims to measure the relationship between personality traits and workplace bullying victims. Moreover, it explores how personality traits predict being a victim of workplace bullying.

Methodology

A cross-sectional study was conducted among 625 participants from various regions of Saudi Arabia. Data was collected using a self-administered survey, which included sociodemographic questions, the Negative Acts Questionnaire-Revised (NAQ-R) for bullying assessment, and the Big Five Personality Inventory (BFI-10) for personality trait assessment. The statistical analysis encompassed descriptive statistics and inferential tests such as the correlation test, Mann-Whitney U test, and Kruskal-Wallis test. These analyses were conducted using the SPSS software version 27.0.1 (IBM Corp., Armonk, NY, USA).

Results

Personal-related bullying was more prevalent compared to work-related bullying, particularly “facing disregard of opinion” was the most frequent type of bullying. Moreover, conscientiousness, agreeableness, and openness to experience were demonstrated as major self-perceived personality traits among participants. Some sociodemographic factors were reported to be significantly associated with both bullying and personality trait scores. Personality traits such as conscientiousness, agreeableness, and extraversion were adversely correlated while openness to experience and neuroticism were positively correlated with bullying.

Conclusions

Our study illustrates the current prevalence of workplace bullying in Saudi Arabia and its impact on participants’ mental health and productivity. We identified a significant correlation between self-perceived personality traits and the risk of experiencing workplace bullying. These findings offer valuable insights for policymakers, enabling them to develop targeted interventions to reduce bullying within work settings in Saudi Arabia.

## Introduction

Workplace bullying is defined as a type of aggressive behavior, wherein these actions are repeated, and it is associated with power differences that make it challenging for the victims of this type of abuse to defend themselves [1,2]. Bullying can include intimidation, public humiliation, and inappropriate teasing [3]. Bullying can come from different people in the work environment, such as supervisors, co-workers, and colleagues. Although these behaviors are sometimes not considered illegal and could be not against workplace policies, they can be destructive for both the victims and the workplace morality [3]. Globally, around 18% of employees have been victims of psychological abuse during their work experience, and more than 8% have encountered physical violence [3]. Workplace bullying can result in increasing rates of absenteeism, stress, depression, feelings of guilt, and decreased productivity, motivation, and morality [4,5]. Working in a detrimental environment where bullying is a common predicament can result in individuals suffering psychological distress as they begin to question their self-esteem and values as well as physical distress or harm [6]. Anxiety, panic attacks, depression, and sleep disorders are some of the devastating mental health issues caused by workplace bullying [7]. Low or lack of self-esteem, feeling impotent, and experiencing suicidal ideations or attempting suicide are all consequences of workplace bullying [8]. In addition to the mental illnesses that result from exposure to bullying in the workplace, there are also physical ailments that victims may suffer from, such as muscle tension, headaches, and digestive issues. According to the latest studies, €239 million is the annual total cost of workplace bullying in Ireland attributable to increased absenteeism amounting to 1.7 million days lost due to exposure to workplace bullying [9]. One contemporary controversy that has arisen from trying to understand why some individuals are more likely to be bullied than others is due to the belief that certain personality traits make some individuals more vulnerable to bullying [10]. This study aims to assess the relationship between personality traits and workplace bullying victims and to understand how some personality traits have an increased risk of being bullied in both healthcare and non-healthcare workplace environments. This study may help in future interventions and in educating the public on how to prevent and protect themselves from this problem. This study aims to contribute to a safer, more respectful, and productive workplace environment.

Study objectives

The main goal of this research is to assess the relationship between personality traits and workplace bullying victims. The specific objectives are to determine how personality traits predict being a victim of workplace bullying, compare the prevalence of workplace bullying in different organizations, and test how it varies according to personality traits. The secondary objective is to identify future potential interventions in helping some people with specific personality traits in how they protect themselves against being victims of bullying.

## Materials and methods

Study area/settings

A cross-sectional study was conducted on the general population in different regions of Saudi Arabia, including both healthcare and non-healthcare settings. The co-investigators collected data using a self-administered questionnaire from December 2023 to March 2024. Table 1 provides an outline of the inclusion and exclusion criteria utilized in this study.

**Table 1:** Inclusion and exclusion criteria.

Inclusion criteria	Exclusion criteria
Male and female	Incomplete data
Saudi and non-Saudi	
Age 18 and above	

Sample size

As the population in Saudi Arabia is estimated to be 35 million, the estimated sample size was calculated to be 385 with a confidence level of 95% and a 5% margin of error.

Sampling technique

In this study, a non-probability convenient sampling technique was used by including all those who met the inclusion criteria.

Data collection methods, instruments used, and measurements

The data was collected through a self-administered questionnaire in Arabic that consisted of three parts. The first was demographic information, including age, gender, education, occupation, income, and a history of any mental disorder. The second part assessed workplace bullying using the Negative Acts Questionnaire-Revised (NAQ-R) [5], which measures how many times the participants have been exposed to negative acts during the last six months and includes 22 items with answers in a 1-5 scale (never, now, monthly, weekly, daily). The third part assessed personality traits using a brief version of the Big Five Personality Inventory 10 (BFI-10) [10]. BFI-10 includes 10 items that measure extraversion, agreeableness, conscientiousness, neuroticism, and openness to experience. Validation and reliability assessments on the questionnaire were conducted by public health experts. Following this, a pilot test was conducted using a diverse small sample from the target population to gather feedback on aspects, such as clarity, length, and comprehensiveness of the questionnaire. Adjustments were made accordingly.

Data management and analysis

Bullying was assessed using the NAQ-R. Each item’s score was aggregated to determine the overall bullying score. Similarly, personality traits were assessed using BFI-10. The analysis involved both descriptive and inferential statistical tests. Simple frequencies and percentages of the categorical variables were calculated and tabulated. For quantitative variables, means, standard deviations (SDs), medians, and interquartile ranges (IQRs) were computed. To find the correlation between various personality traits and bullying scores, the Spearman rho correlation test was employed. Furthermore, the Mann-Whitney U test and the Kruskal-Wallis test were utilized to compare bullying scores and personality trait scores among different sociodemographic characteristics. These non-parametric tests were chosen owing to the non-normal distribution of the variables calculated using the Shapiro-Wilk test. Statistical significance was established at a p-value of 0.05 or less with a 95% confidence interval. All statistical calculations were performed using the SPSS Software version 27.0.1 (IBM Corp., Armonk, NY, USA). There were a total of 625 responses.

The co-investigators ensured that patients understood their participation was voluntary and their responses would remain confidential. The questionnaire was distributed among participants as a self-administered questionnaire, with the entire process requiring less than 10 minutes to complete. Additionally, the questionnaires were translated into Arabic and subjected to expert back-translation to ensure accuracy. All information was securely maintained without gathering any identifying details, such as MRN, names, or ID (MRN replaced with a serial number), with no collection of identifiers, and strict storage protocols within NGHA premises for both physical and digital records. Access to research data was restricted to research group members only. The study received ethical approval from the Institutional Review Board of King Abdullah International Medical Research Center, Ministry of National Guard Health Affairs, Riyadh, Kingdom of Saudi Arabia (approval number: IRB/1846/23).

## Results

Table 2 summarizes the sociodemographic characteristics of 625 participants, with a balanced gender distribution (51.2% males, 48.8% females) and an average age of 34.2 years. Regionally, most participants were from the Eastern Province (48.3%), followed by the Central Region (37.0%) and the Western Region (14.7%). Marital status showed a majority of singles (52.0%) and married individuals (44.5%). Education levels revealed that 73.6% had completed university education, and employment status indicated that 52.0% were employees, with 64.5% in governmental positions. In the healthcare sector, 21.8% worked, with doctors comprising the largest group (67.0%). Monthly income distribution showed 53.1% of participants earning less than 10,000 SAR, and 73.7% reported no additional income sources. Mental health data indicate that 12.0% reported a diagnosis, 47.4% preferred not to disclose, and 40.6% reported no diagnosed mental disorder.

**Table 2:** Sociodemographic characteristics of participants (n = 625). n: frequency; %: percentage. SD: standard deviation; SAR: Saudi riyal

Variable		n (%)
Age (mean ± SD)	34.2 ± 13.5	
Gender	Male	320 (51.2%)
	Female	305 (48.8%)
Region	Central Region	231 (37.0%)
	Eastern Province	302 (48.3%)
	Western Region	92 (14.7%)
Marital status	Single	325 (52.0%)
	Married	278 (44.5%)
	Divorced	17 (2.7%)
	Widowed	5 (0.8%)
Education level	Intermediate	6 (1.0%)
	Secondary	91 (14.6%)
	University	460 (73.6%)
	Master’s	45 (7.2%)
	PhD	23 (3.7%)
Employment status	Student	155 (24.8%)
	Employee	325 (52.0%)
	Unemployed	62 (9.9%)
	Retired	83 (13.3%)
Sector of the job	Governmental	403 (64.5%)
	Non-governmental	222 (35.5%)
Within/Outside the healthcare system	Outside the healthcare sector	489 (78.2%)
	Within the healthcare sector	136 (21.8%)
Your job within the healthcare system	Doctor	73 (67.0%)
	Nurse	10 (9.2%)
	Specialist	14 (12.8%)
	Pharmaceutical	12(11.0%)
	Doctor	73 (67.0%)
Role of doctors	Intern doctor	50 (64.1%)
	Resident doctor	9 (11.5%)
	General doctor	10 (12.8%)
	Deputy doctor	2 (2.6%)
	Consultant doctor	7 (9.0%)
Monthly income	Less than 10,000 SAR	332 (53.1%)
	10,000–20,000 SAR	178 (28.5%)
	20,000–30,000 SAR	63 (10.1%)
	More than 30,000 SAR	52 (8.3%)
Other sources of income	No	426 (73.7%)
	Yes	152 (26.3%)
Diagnosed with any mental disorder	Yes	75 (12.0%)
	No	254 (40.6%)
	Prefer not to say	296 (47.4%)

Table 3 provides a concise overview of the descriptive statistics for both bullying scores and self-perceived personality traits within the participant pool. On average, participants reported a total bullying score of 36, with an SD of 14. Further disaggregation of the data revealed that work-related bullying had an average score of 13, personal-related bullying demonstrated an average score of 19, and physically intimidating bullying garnered an average score of 4. Regarding personality traits, participants indicated varying levels of self-perception. Extraversion exhibited an average score of 6, agreeableness garnered an average score of 7, conscientiousness yielded an average score of 8, and neuroticism displayed an average score of 6. Lastly, openness to experience demonstrated an average score of 7 with an SD of 2.

**Table 3:** Descriptive statistics of bullying and personality trait scores. SD: standard deviation

	Mean ± SD
Total bullying scores	36 ± 14
Work-related bullying (7 items)	13 ± 5
Personal-related bullying (12 items)	19 ± 8
Physically intimidating bullying (3 items)	4 ± 2
Personality traits	
Extraversion (2 items)	6 ± 2
Agreeableness (2 items)	7 ± 2
Conscientiousness (2 items)	8 ± 2
Neuroticism (2 items)	6 ± 2
Openness to experience (2 items)	7 ± 2

Table 4 provides a comprehensive account of participants’ responses to NAQ-R, revealing the prevalence of various forms of workplace bullying. A significant proportion (45.8%) stated that they had never experienced someone withholding information affecting their performance, while 43.8% reported occasional instances of such behavior. Regarding tasks below their competence, the majority (51.4%) reported experiencing this sometimes, with 31.5% stating they had never encountered it. Ignoring opinions and suggestions was reported by 54.2% occasionally, while 28.5% stated they had never experienced this behavior. A significant majority (71.2%) reported that they had never experienced any hints or signals from others that they should leave their job, while 20.3% stated they had experienced this sometimes. More than half of the participants (54.9%) reported experiencing frequent reminders of their mistakes sometimes, while 33.9% stated they had never experienced it. A smaller percentage reported monthly (5.3%), weekly (2.7%), or daily (3.2%) occurrences. A vast majority (89.1%) reported never experiencing threat of violence, physical harm, or actual harm, while 7.8% stated they had experienced such behavior sometimes. A smaller percentage reported monthly (1.1%), weekly (0.8%), or daily (1.1%) occurrences.

**Table 4:** Participants’ responses regarding bullying. n: frequency; %: percentage.

Question	Never	Sometimes	Monthly	Weekly	Daily
	n (%)	n (%)	n (%)	n (%)	n (%)
Someone is withholding information that affects your performance	286 (45.8%)	274 (43.8%)	20 (3.2%)	20 (3.2%)	25 (4.0%)
Being asked to do work below your level of competence	197 (31.5%)	321 (51.4%)	35 (5.6%)	42 (6.7%)	30 (4.8%)
Ignore your opinions and suggestions	178 (28.5%)	339 (54.2%)	39 (6.2%)	41 (6.6%)	28 (4.5%)
Assigning tasks with unreasonable deadlines	269 (43.0%)	249 (39.8%)	52 (8.3%)	32 (5.1%)	23 (3.7%)
Excessive monitoring of your work	271 (43.4%)	218 (34.9%)	39 (6.2%)	42 (6.7%)	55 (8.8%)
Pressure not to claim something you are entitled to	345 (55.2%)	206 (33.0%)	36 (5.8%)	15 (2.4%)	23 (3.7%)
Exposure to uncontrollable workload	239 (38.2%)	274 (43.8%)	45 (7.2%)	33 (5.3%)	34 (5.4%)
Being insulted or ridiculed in relation to your work	418 (66.9%)	152 (24.3%)	27 (4.3%)	13 (2.1%)	15 (2.4%)
Remove key areas of responsibility or replace them with trivial or unpleasant tasks	349 (55.8%)	209 (33.4%)	25 (4.0%)	22 (3.5%)	20 (3.2%)
Gossip and rumors about you	285 (45.6%)	248 (39.7%)	33 (5.3%)	24 (3.8%)	35 (5.6%)
Ignoring or excluding	300 (48.0%)	242 (38.7%)	31 (5.0%)	17 (2.7%)	35 (5.6%)
Make offensive or offensive remarks about you	343 (54.9%)	211 (33.8%)	28 (4.5%)	19 (3.0%)	24 (3.8%)
Hints or signals from others that you should leave your job	445 (71.2%)	127 (20.3%)	22 (3.5%)	14 (2.2%)	17 (2.7%)
Frequent reminders of your mistakes	343 (54.9%)	212 (33.9%)	33 (5.3%)	17 (2.7%)	20 (3.2%)
Being ignored or facing a hostile reaction when trying to get closer	404 (64.6%)	172 (27.5%)	19 (3.0%)	12 (1.9%)	18 (2.9%)
Constant criticism for your mistakes	366 (58.6%)	194 (31.0%)	31 (5.0%)	17 (2.7%)	17 (2.7%)
Certain jokes made by people you don’t get along with	359 (57.4%)	196 (31.4%)	32 (5.1%)	20 (3.2%)	18 (2.9%)
Exposure to the charges against you	416 (66.6%)	158 (25.3%)	23 (3.7%)	16 (2.6%)	12 (1.9%)
Exposure to excessive harassment and ridicule	443 (70.9%)	135 (21.6%)	19 (3.0%)	14 (2.2%)	14 (2.2%)
Yelling at you or being a constant target for venting anger	458 (73.3%)	119 (19.0%)	22 (3.5%)	12 (1.9%)	14 (2.2%)
Scary behavior such as pointing fingers	471 (75.4%)	115 (18.4%)	21 (3.4%)	9 (1.4%)	9 (1.4%)
The threat of violence, physical harm, or actual harm	557 (89.1%)	49 (7.8%)	7 (1.1%)	5 (0.8%)	7 (1.1%)

Table 5 presents participants’ responses to BFI‐10, which assesses various personality traits. The findings indicated a diverse range of self-perceptions within the sample. A significant proportion (30.4%) slightly agreed that they were conservative, while 28.3% remained neutral. Regarding general trust in others, responses were fairly balanced, with 28.6% slightly agreeing and 25.8% remaining neutral. Social inclinations were prevalent, as 32.6% strongly agreed, and an additional 30.9% slightly agreed while 7.8% strongly disagreed. Attention to detail was notably emphasized, with 48.3% strongly agreeing. Stress susceptibility was acknowledged by 32.3%, while 17.8% strongly agreed. Imagination was regarded highly, with 37.4% strongly agreeing.

**Table 5:** Participants response regarding personality traits (BFI‐10) (n = 625). n: frequency; %: percentage. BFI‐10: Big Five Personality Inventory

	Strongly disagree	Slightly disagree	Neutral	Slightly agree	Strongly agree
	n (%)	n (%)	n (%)	n (%)	n (%)
I am conservative	51 (8.2%)	72 (11.5%)	177 (28.3%)	190 (30.4%)	135 (21.6%)
I trust others in general	97 (15.5%)	124 (19.8%)	161 (25.8%)	179 (28.6%)	64 (10.2%)
I tend to be lazy	233 (37.3%)	144 (23.0%)	96 (15.4%)	118 (18.9%)	34 (5.4%)
I am calm and deal with pressure well	45 (7.2%)	88 (14.1%)	109 (17.4%)	203 (32.5%)	180 (28.8%)
I have few interests in art	136 (21.8%)	97 (15.5%)	130 (20.8%)	160 (25.6%)	102 (16.3%)
I am social	49 (7.8%)	68 (10.9%)	111 (17.8%)	193 (30.9%)	204 (32.6%)
I tend to look for faults in others	347 (55.5%)	129 (20.6%)	75 (12.0%)	57 (9.1%)	17 (2.7%)
I do the work accurately and pay attention to details	19 (3.0%)	49 (7.8%)	68 (10.9%)	187 (29.9%)	302 (48.3%)
I get stressed easily	80 (12.8%)	98 (15.7%)	111 (17.8%)	202 (32.3%)	134 (21.4%)
I have a great imagination	52 (8.3%)	55 (8.8%)	105 (16.8%)	179 (28.6%)	234 (37.4%)

Table 6 presents a comprehensive comparison of total bullying scores across various sociodemographic characteristics, revealing potential associations. Gender, region-based differences, education level, and monthly income did not significantly impact bullying scores. However, marital status significantly influenced bullying scores (p = 0.004). Divorced individuals reported the highest median score (38), followed by widowed (35), single (33), and married participants (31). Post hoc tests confirmed differences between single and married as well as between married and divorced individuals. Employment status also significantly impacted scores (p = 0.002). Unemployed individuals reported the highest median (35), followed by employees, students, and retired individuals. Post hoc tests confirmed differences between student and retired, employee and retired, and unemployed and retired participants. The top-form sector of the job showed a statistically significant difference in scores (p = 0.002), with non-governmental sector participants reporting a slightly higher median score of 34 compared to those in the governmental sector with a median score of 32. Job roles within the healthcare system significantly differed in bullying scores (p = 0.005). Specialists had the highest median score (48), followed by pharmaceutical professionals (43), nurses (34), and doctors (32). Post hoc tests confirmed significant differences between doctors and specialists, as well as between nurses and specialists. Participants reporting a diagnosis with a mental disorder had a higher median score of 34 compared to those without a diagnosis (median score of 30), with the difference being statistically significant (p < 0.001).

**Table 6:** Comparison of total bullying scores among different sociodemographic characteristics. U: Mann-Whitney U test; H: Kruskal-Wallis test; *: p < 0.05, significant. IQR: interquartile range; SAR: Saudi riyal

Variable		Total bullying score		Significance
Age^ ρ^ (r = -0.117)				0.003
		Median	IQR	
Gender	Male	32	27–42	0.575^U^
	Female	32	28–42	
Region	Central Region	32	27–40	0.429^H^
	Eastern Province	32	27–43	
	Western Region	33	27–43	
Marital status	Single	33	28–44	0.004*^H^
	Married	31	26–39	
	Divorced	38	30–47	
	Widowed	35	22–89	
Education level	Intermediate	28	26–30	0.301^H^
	Secondary	31	26–42	
	University	32	27–42	
	Master’s	31	28–37	
	PhD	38	26–49	
Employment status	Student	32	28–42	0.002*^H^
	Employee	32	27–43	
	Unemployed	35	26–45	
	Retired	30	25–34	
Sector of the job	Governmental	32	26–40	0.002*^U^
	Non-governmental	34	28–44	
Within/Outside the healthcare system	Outside the healthcare sector	32	27–41	0.336^U^
	Within the healthcare sector	33	28–43	
Your job within the healthcare system	Doctor	32	27–42	0.005*^H^
	Nurse	34	31–38	
	Specialist	48	37–70	
	Pharmaceutical	43	32–32	
Role of doctors	Intern doctors	32	26–42	0.802^H^
	Resident doctor	32	23–44	
	General doctor	39	31–44	
	Deputy doctor	55	26–83	
	Consultant doctor	31	24–52	
Monthly income for the job	Less than 10,000 SAR	33	28–44	0.055^H^
	10,000–20000 SAR	32	27–40	
	20,000–30,000 SAR	30	25–38	
	More than 30,000 SAR	34	28–40	
Other sources of income	Yes	32	27–43	0.762^U^
	No	32	27–42	
Diagnosed with any mental disorder	Yes	34	29–44	<0.001*^H^
	No	30	26–39	
	Preferred not to say	34	27–44	

Table 7 unveils intriguing patterns in total bullying scores across sociodemographic characteristics. Gender differences were significant (p = 0.002), with males scoring higher compared to females. Regionally, scores showed no substantial variations (p = 0.608) across Central, Eastern, and Western provinces. Marital status significantly impacted scores (p < 0.001), with divorced individuals exhibiting the highest scores and widowed participants the lowest. Education levels showed no prominent differences (p = 0.602). Employment status significantly influenced scores (p < 0.001), with retirees displaying the highest scores and students the lowest. Job sector differences were notable (p = 0.026), with those working within the healthcare sector scoring slightly lower. Healthcare roles showed subtle differences (p = 0.190). Monthly income, other sources of income, and a diagnosis of a mental disorder did not lead to substantial differences in total personality trait scores.

**Table 7:** Comparison of total personality trait scores among different sociodemographic characteristics. U: Mann-Whitney U test; H: Kruskal-Wallis test; *: p < 0.05, significant. IQR: interquartile range; SAR: Saudi riyal

		Total personality trait score		Significance
		Median	IQR	
Gender	Male	34	32–36	0.002*^U^
	Female	33	31–36	
Region	Central Region	33	31–36	0.608^H^
	Eastern Province	34	31–36	
	Western Region	34	31–37	
Marital status	Single	33	31–35	<0.001*^H^
	Married	34	32–37	
	Divorced	36	33–37	
	Widowed	29	25–29	
Education level	Intermediate	33	32–35	0.602^H^
	Secondary	34	31–37	
	University	33	31–36	
	Master’s	35	33–37	
	PhD	35	34–38	
Employment status	Student	33	30–35	<0.001*^H^
	Employee	34	32–37	
	Unemployed	33	31–35	
	Retired	35	32–37	
Sector of the job	Governmental	34	31–36	0.756^U^
	Non-governmental	33	31–36	
Within/Outside healthcare system	Outside the healthcare sector	34	31–36	0.026*^U^
	Within the healthcare sector	33	31–35	
Your job within the healthcare system	Doctor	33	32–35	0.256^H^
	Nurse	32	30–34	
	Specialist (graduate of applied medical sciences)	34	32–37	
	Pharmaceutical	32	31–35	
Role of doctors:	Intern doctors	33	31–35	0.190^H^
	Resident doctor	33	32–34	
	General doctor	34	32–37	
	Deputy doctor	37	33–40	
	Consultant doctor	35	34–35	
Monthly income for the job	Less than 10,000 SAR	33	31–36	0.149^H^
	10,000–20,000 SAR	34	32–37	
	20,000–30,000 SAR	33	31–36	
	More than 30,000 SAR	34	32–35	
Other sources of income	Yes	34	31–35	0.260^U^
	No	34	31–36	
Diagnosed with any mental disorder	Yes	34	32–36	0.172^H^
	No	34	32–36	
	Preferred not to say	33	31–36	

Table 8 provides insights into the correlations between personality traits and bullying scores. Higher extraversion scores showed a weak negative correlation with bullying (ρ = -0.088, p = 0.027), suggesting a lower likelihood of experiencing bullying. Agreeableness scores demonstrated a negative correlation (ρ = -0.143, p < 0.001), indicating reduced likelihood of bullying. Conscientiousness scores revealed a negative correlation (ρ = -0.119, p = 0.003), implying lower susceptibility to bullying. Conversely, higher neuroticism scores showed a notable positive correlation (ρ = 0.201, p < 0.001), indicating an increased likelihood of experiencing bullying. Openness to experience scores exhibited a weak positive correlation (ρ = 0.090, p = 0.024), suggesting a slight increase in likelihood. All these correlations were statistically significant.

**Table 8:** Correlation between personality traits and bullying scores ρ: Spearman rho correlation; *: p < 0.05, significant.

Personality trait	Bullying score	
	Correlation coefficient ^ρ^	Significance
Extraversion score	-0.088	0.027*
Agreeableness score	-0.143	<0.001*
Conscientiousness score	-0.119	0.003*
Neuroticism score	0.201	<0.001*
Openness to experience score	0.090	0.024*

## Discussion

Workplace bullying is defined as repeated inappropriate behavior that is persistently shown to the victim at the workplace by a bullying individual or a group of individuals [11]. Workplace bullying involves a variety of insulting or aggressive behaviors such as verbal bullying, physical abuse, or sexual harassment. Workplace bullying can occur in any industry, and it can lead to a considerable impact on the victims’ mental as well as physical health [6]. A study reported that bullying has been linked to increased rates of workforce absence from healthcare centers in Ireland causing a loss of 1.7 million working days costing €239 million [9]. Furthermore, some personality traits might be associated with a higher risk of being bullied in the workplace [10]. In the healthcare work environment, bullying has been reported with varying prevalence and multiple associated factors in different studies. This study was conducted to assess the relationship between personality traits and the associated risk of bullying in individuals with certain characteristics in the workplace environment.

The current study was conducted following a cross-sectional design and recruited data from more than 600 participants, equally distributed for gender (51.2% males, 48.8% females). The average age of the participants was 34.2 years, suggesting that the majority of the working population is middle-aged. The majority of the participants belonged to the Eastern Province of Saudi Arabia (48.3%) while the least number of participants were from the Western Region (14.7%), indicating the Eastern population a potential target population for implementing strategies to reduce the risk of workplace bullying. Our findings also depicted that the majority (73.6%) of our studied population was well-educated (university graduates) and held government positions (64.5%) while the majority (67%) were serving as doctors. Despite that, almost half of our studied population (47.4%) chose not to disclose their status about a diagnosis of mental health. These findings are in accordance with previously reported studies signifying that mental health issues are considered a stigma even in well-educated sections of society in Saudi Arabia [12].

The analysis of perceived personality traits and descriptive statistics of bullying reported by study participants depicted that personal-related bullying was higher in our study cohort compared to work-related bullying. However, Rahm et al. (2019) showed work-related bullying to be higher in the Swedish healthcare environment when compared to person-related bullying [13]. In addition, conscientiousness was the most prominent personality trait in our study cohort while neuroticism and extraversion were least prevalent. These outcomes were different from a previously published study conducted by Shehzad et al. (2020) describing conscientiousness as the least frequent while neuroticism and extraversion as more prevalent personality traits in Pakistani medical students [14].

Moreover, our data analysis revealed that workplace bullying in the currently studied population is a prevalent phenomenon, consistenct with the previously reported 63.7% in a Saudi study cohort of more than 1000 healthcare workers [11]. A recent study from Saudi Arabia reported that 26.6% of healthcare workers reported experiencing workplace bullying [15]. The most common forms of workplace bullying included ignoring opinions and disregarding suggestions of participants (71.5%) and assignments of tasks below competence (68.5%). These outcomes were similar to a previous study published by Rouse et al. (2016) wherein 72.9% of physicians in the United States healthcare centers reported being bullied as their opinions were disregarded [16].

The results of our BFI-10 survey indicated a diverse range of self-perceptions within the sample, for instance, 52% of the participants perceived themselves to be conservative while 28.3% remained neutral. It suggests that openness and extraversion were not frequently perceived personality traits in our studied population. However, conscientiousness and agreeableness were depicted as major self-perceived personality traits in our study sample. The BFI-10 survey results reveal some differences and similarities between our sample and other samples from previously published resources in their self-reported personality traits, indicating that BFI-10 may prove to be an efficient tool for measuring human personality traits [17].

Furthermore, we also explored the association between various sociodemographic factors and bullying scores in our studied population. The results showed that marital status, employment status, job sector, job role, and mental disorder diagnosis significantly influenced the bullying scores, while gender, region, education level, and monthly income did not. However, a previous study by Al-Surimi et al. (2020) reported that workplace bullying was significantly associated with gender and educational level [11]. Previously, a nationwide cross-sectional study by Serafin and Czarkowska-Pączek (2019) in Poland found that age, seniority level, overtime in work, and having a bachelor’s degree were majorly associated factors of bullying faced by nurses [18]. Moreover, our results contradicted those of a previous study from Saudi Arabia which analyzed data from healthcare workers and observed significant correlations between workplace bullying and sociodemographic parameters, including gender, age, nationality, and education level [11]. Our findings and previous literature suggest that the impact of sociodemographic factors on bullying experiences may vary depending on the context, culture, and sample of healthcare workers.

In addition, we assessed total personality scores and their association with sociodemographic parameters in our study population. Our findings suggested that gender, marital status, employment status, and job within/outside the healthcare sector were significantly related to the total personality trait scores in our study cohort. These outcomes were similar to those published by Khubchandani and Price (2017) in a cross-sectional study conducted among US workers and described gender, race, and marital status to be associated with workplace bullying [19].

Finally, we observed various significant correlations between the personality traits of the study participants and bullying scores. For instance, we observed that conscientiousness, agreeableness, and extraversion had a negative correlation with bullying. It suggested that study participants with higher scores in these personality traits were less likely to be the victims of workplace bullying. On the other hand, there was a positive correlation between the risk of being bullied at the workplace and having personality traits such as openness to experience and neuroticism. Hence, participants with higher scores in neuroticism and openness to experience are more likely to get bullied at their workplace. These results were similar to a previous study in which Kulig et al. (2018) reported a positive correlation between neuroticism and being a victim of bullying among a sample of more than 2,000 adolescent school students in the United States [20]. Similarly, neuroticism was found positively associated with a higher risk of workplace bullying when data was analyzed from a cohort of junior doctors working in the healthcare sector of Malaysia [21]. In addition, our findings about correlations between bullying risk and personality traits, including conscientiousness, agreeableness, and extraversion, were consistent with the outcomes of a study conducted by Rai and Agarwal (2019) by analyzing a sample population of Indian managers. However, their results differ from ours regarding openness to experience as they also found a negative correlation between openness to experience and the risk of bullying among study participants [22]. In addition, our study outcomes were consistent with the findings of Ramaci et al. (2020), who depicted a negative correlation between workplace bullying and conscientiousness and agreeableness in an Italian cohort of healthcare workers [23].

Limitations

Although the current study provided meaningful insights into a relatively less explored subject in the given population, it is important to acknowledge the limitations that may have impacted the results of this study. First, the study followed a cross-sectional design which cannot establish cause-effect relationships. Second, our findings were based on self-reported data which is likely to affect the reliability of outcomes as responses are often biased or inaccurate. Finally, the study sample was selected using a non-probability convenient sampling technique which may add bias to the samples, lack variety, and limit external validity.

## Conclusions

Our study provides valuable insights into the potential factors impacting the productivity of the workforce in Saudi Arabia. Our study outcomes highlighted that there is considerable workplace bullying in Saudi Arabia and may impact the mental health of the studied population. Moreover, our results depicted correlations between the risk of workplace bullying and self-perceived personality traits in the population of Saudi Arabia. These findings may provide potential targets for the design and implementation of interventional approaches focused on reducing the risk of workplace bullying in Saudi Arabia.
